# Correlation between Inflorescence Architecture and Floral Asymmetry—Evidence from Aberrant Flowers in *Canna* L. (Cannaceae)

**DOI:** 10.3390/plants11192512

**Published:** 2022-09-26

**Authors:** Qianxia Yu, Tong Zhao, Haichan Zhao, Chelsea D. Specht, Xueyi Tian, Jingping Liao

**Affiliations:** 1 Department of Grassland Science, College of Forestry and Landscape Architecture, South China Agricultural University, Guangzhou 510642, China; 2 Key Laboratory of Plant Resources Conservation and Sustainable Utilization, South China Botanical Garden, Chinese Academy of Sciences, Guangzhou 510650, China; 3 Guangdong Eco-Engineering Polytechnic, Guangzhou 510520, China; 4 Guangdong Yunfu Vocational College of Chinese Medicine, Yunfu 527400, China; 5 School of Integrative Plant Science, Section of Plant Biology and the L.H. Bailey Hortorium, Cornell University, Ithaca, NY 14853, USA; 6 Foshan Institute of Forestry, Foshan 528222, China

**Keywords:** aberrant flower, asymmetric flower, *Canna*, floral symmetry, inflorescence, Zingiberales

## Abstract

Floral symmetry studies often focus on the development of monosymmetric and polysymmetric flowers, whereas asymmetric flowers and their position and function within the inflorescence structure are largely neglected. Cannaceae is one of the few families that possesses truly asymmetric flowers, serving as a model to study the characters and mechanisms involved in the development of floral asymmetry and its context within the developing and mature inflorescence. In this study, inflorescence structure and floral morphology of normal asymmetric flowers and 16 aberrant flower collections from *Canna indica* L. and *C. glauca* L. were photographed, analyzed, and compared with attention to stamen petaloidy, floral symmetry, and inflorescence branching patterns anterior and posterior to the aberrant flower. In comparison with normal flowers, the aberrant flowers are arranged into abnormal partial florescences, and vary in floral symmetry, orientation, and degree of androecial petaloidy. The appendage of the fertile stamen is universally located distal from the higher order bract, indicating an underlying influence of inflorescence architecture. A synthetic model is proposed to explain the relationship between floral symmetry and inflorescence structure. Data from the observation of aberrant phenotypes strongly support the hypothesis that irregular petaloidy of the stamens is correlated with an asymmetric morphogenetic field within the inflorescence that contributes to the overall floral asymmetry in *Canna* flowers.

## 1. Introduction

The monocot family Cannaceae (Zingiberales) comprises ten species distributed throughout the Neotropics, which are all placed in the single genus *Canna* L. (Figure S1) [1,2]. Cannaceae species are widely cultivated as ornamental plants and they play a significant role in the medicinal and food industry.

In zygomorphic (monosymmetric) and asymmetric flowers found throughout the angiosperms, floral symmetry patterns are often the results of the elaboration and/or abortion of distinct floral organs [3,4]. The asymmetry of *Canna* flowers is primarily due to the irregular petaloidy among the androecial members, a fertile stamen, and several staminodes, which dominate the floral display. *Canna* flowers comprise two trimerous whorls of fertile stamen and staminodes. In the outer androecial whorl of most species, the lateral staminode develops into a laminar (petaloid) floral organ, whereas the other two staminodes abort soon after initiation [5,6]. The inner whorl consists of a curved staminode often with colorful markings called the ‘labellum’, a petaloid staminode, and a single fertile stamen which is only ½ fertile, bearing a single theca with an expanded petaloid appendage [5,6,7,8,9,10,11]. The half-fertile stamen is thought to be associated with secondary pollen presentation [12]. Both the labellum and the ½ fertile stamen are asymmetric organs. The style is laminar with a linear stigmatic surface extending down one side [13], making the gynoecium also appear asymmetric. The actinomorphy of the perianth combined with the asymmetry of the androecial and gynoecial whorls and the organs therein makes *Canna* an ideal taxon to study the elaboration of floral asymmetry.

There is a well-documented correlation between floral symmetry and inflorescence architecture [14,15,16,17]. Solitary flowers which are not part of an inflorescence are generally actinomorphic (i.e., polysymmetric), as seen in Magnoliaceae, Ranunculaceae, and Rosaceae species. Axillary flowers in a racemose inflorescence (e.g., raceme, spike and spadix) are typically zygomorphic at initiation, even though some may develop into actinomorphic mature flowers [18]. In the Zingiberales, examples of this correlation are prominent in the families of Strelitziaceae and Heliconiaceae, in which flowers are not strictly oriented toward the main inflorescence axis and are influenced by neighboring structures in the cincinnus. In *Phenakospermum* Endl. (Strelitziaceae), the flowers are oriented so that their median plane passes between the next-older flower and the inflorescence axis, whereas in *Heliconia* L. (Heliconiaceae), the median plane of the flower bisects the next-older flower. In both *Phenakospermum* and *Heliconia*, adjacent flowers are mirror images of each other [19]. 

Floral symmetry can also vary among flowers within an inflorescence: for example, different floral symmetry types are found within the capitulum inflorescence of many Asteraceae and Dipsacaceae species. In *Gerbera* L. (Asteraceae), three different types of flowers (ray [zygomorphic], trans [slightly zygomorphic], and disk [polysymmetric]) are positioned along the radial axis of the capitulum [20]. In the *centroradialis* (*cen*) mutants of *Antirrhinum* L., the inflorescence changes from an open to a closed raceme, forming a terminal flower at the top. The terminal flower (or flower-like terminal structure) is actinomorphic and thus differs in symmetry from the zygomorphic lateral flowers [14]. Terminal flowers have been observed as naturally occurring variants in a wide range of species [21,22,23]. Whether occurring spontaneously or induced by mutation, the actinomorphic terminal flower suggests that the symmetry of flowers is linked to their position within an inflorescence. 

Inflorescence architecture is diverse across angiosperms and can involve multiple orders of branching, each order with its own underlying topology. Inflorescences are classified based on order and position of branching and terminal units, composition of terminal units, differential elongation of axes of different orders, and repetition of basic ramification patterns across orders [24]. The terminology of inflorescences is heavily typological and sometimes ambiguous, making it difficult to model inflorescence architecture and to define homologies across inflorescence types. This in turn makes it challenging to clarify functional and evolutionary relationships between inflorescence architecture and floral symmetry across the angiosperms [15,25].

In this study, we use the diversity of *Canna* flowers in a lineage-based approach to explore the relationship between floral symmetry and inflorescence architecture. Aberrant flowers found in *C. indica* L. and *C. glauca* L. are documented, their patterns of floral organ organization and floral symmetry are characterized, and the architecture of the partial florescence in which the flowers occur are identified and described. A general rule revealed by this study explains the correlation between the pattern of androecial petaloidy and the branching system of the inflorescence. These data help define developmental patterns and constraints associated with floral organ formation and potentially reveal the origin of overall asymmetry in *Canna* flowers from a morphological perspective.

## 2. Materials and Methods

### 2.1. Plant Material

Two species of Cannaceae were examined for flower and inflorescence characteristics: *C. indica* and *C. glauca* (Figure S1) [2]. All flowers, including normal and aberrant flowers, were collected from the South China Botanical Garden (SCBG), Chinese Academy of Sciences (CAS), Guangzhou, during February to May 2018. The *C. indica* plants were originally introduced as rhizomes from Beijing Botanical Garden, Beijing, China (5 June 2004) and cultivated in the Ginger Garden of SCBG (living collection accession number: 20150507). The *C. glauca* plants were purchased from a market and cultivated in the Aquatic Plants Garden of SCBG without official accession. Nearly one hundred individuals were propagated from these accessions, and more than one thousand partial florescences were observed with the majority of them bearing cincinni and flowers with the normal (wild type) phenotype. A total of 16 aberrant flowers were observed and collected for further study (Table 1). For each species, eight samples were examined. Each collection record refers to a partial florescence (PF). Except for Ci-7 and Ci-8 which are collected from the same synflorescence, every other aberrant partial florescence is collected from a unique synflorescence.

### 2.2. Morphological Analysis

**Figure 1:**
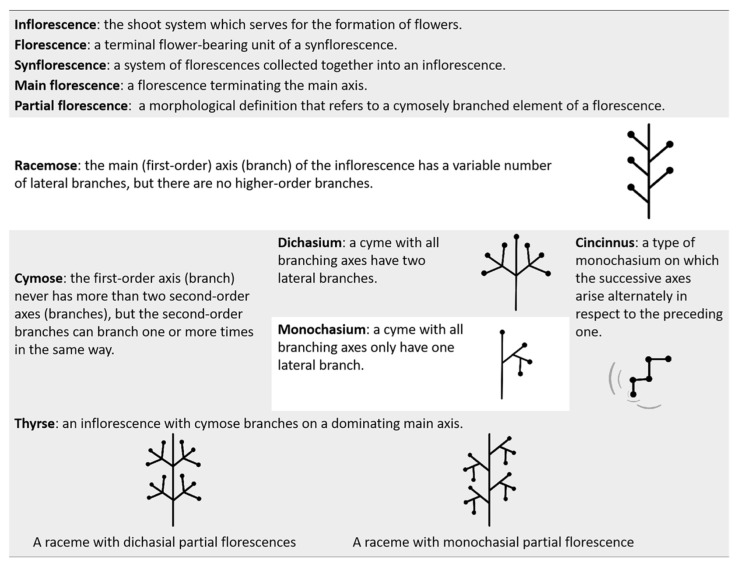
Terminology of inflorescence morphology.

Inflorescences and flowers were dissected and observed immediately following harvest. Photographs were taken with a Sony α7 Camera (Sony, Tokyo, Japan), and all images were adjusted and incorporated using Adobe Photoshop CC 2018. Sketches of inflorescences and floral diagrams were made in Adobe Illustrator CC 2018 using designated symbols for each organ (Figure 1).

Inflorescence structure of the aberrant collections was determined by examining bract placement. The position of a higher order inflorescence meristem was determined by the presence of a subtending bract, and is indicated by a small black dot (transverse view) or an arrow (longitudinal view) for each partial florescence in the illustration. The labellum was distinguished from other petaloid staminodes by the presence of yellow spots and the distinctive curved shape. 

### 2.3. Terminology

**Figure 2:**
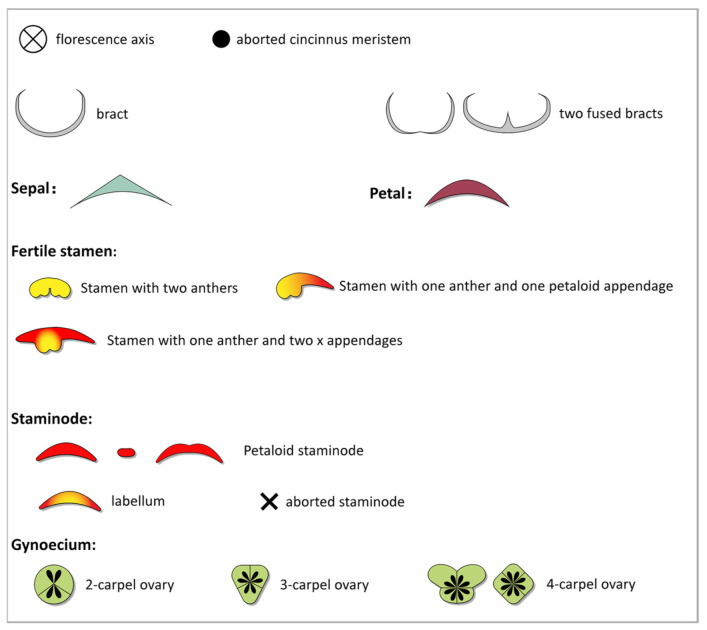
Symbols and their descriptions for floral diagrams.

The terminology of *Canna* inflorescence elements (Figure 1) and floral organs (Figure 2) follows previous literature [5,24,26,27]. 

## 3. Results

### 3.1. Morphology of Normal Canna Inflorescence and Flower

**Figure 3:**
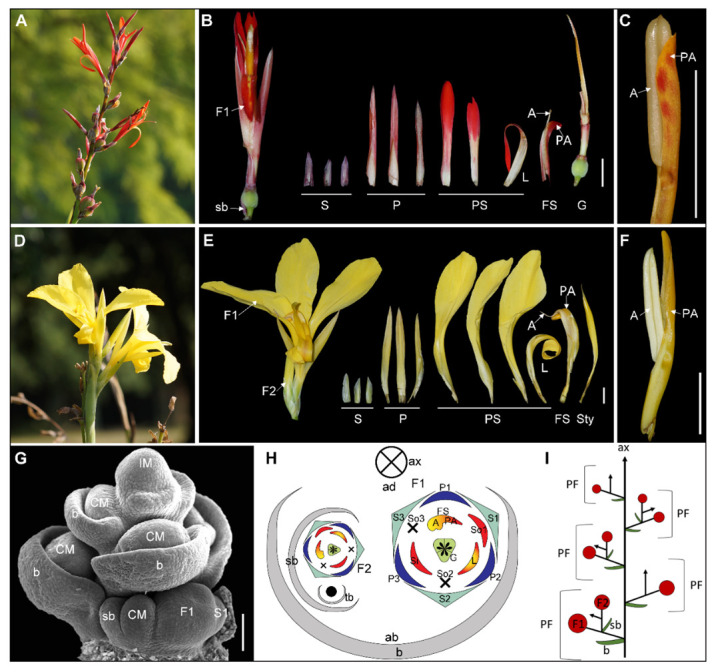
Normal type of inflorescence and flower in *Canna*. (**A**) to (**C**) *C. indica*. (**A**) Inflorescence of *C. indica*; (**B**) 1-flowered cincinnus and flower dissection of *C. indica*; (**C**) The fertile stamen of *C. indica*. (**D**) to (**F**) *C. glauca*; (**D**) Inflorescence of *C. glauca*; (**E**) 2-flowered cincinnus and flower dissection of *C. glauca*; (**F**) The fertile stamen of *C. glauca*; (**G**) SEM picture shows a developing inflorescence of *C. indica*; (**H**) Diagram showing the ground plan of partial inflorescence of normal type *Canna*; (**I**) Illustration of *Canna* inflorescence from lateral view. A, anther; ax, main florescence axis; b, primary bract; CM, cincinnus meristem; F1, the primary flower; F2, the secondary flower; FS, fertile stamen; G, gynoecium; IM, main inflorescence meristem; L, labellum; P, petal; P1, adaxial petal; P2, abaxial petal; P3, lateral petal; PA, petaloid appendage; PF, partial inflorescence; PS, petaloid staminode; S, sepal; S1, lateral sepal; S2, abaxial sepal; S3, adaxial sepal; sb, the secondary bract; Si, inner petaloid staminode; So1, lateral outer petaloid staminode; So2, aborted abaxial outer staminode; So3, aborted adaxial outer staminode; Sty, style; tb, the tertiary bract. The Bars: (**B**,**C**,**E**,**F**) = 1 cm; (**G**) = 100 μm.

The *Canna* inflorescence can be described as a sympodial thyrse or ‘bracteate raceme of cymes’ (Figure 3A,D), which consists of a single main florescence axis (ax) and many partial florescences (PF), each subtended by a primary bract (b; Figure 3G,H). Each partial florescence is a cincinnus (Figure 3H,I). Primary bracts are formed acropetally in a spiral pattern along the main florescence axis. A cincinnus meristem (CM) is initiated in the axil of each primary bract (Figure 1G). Each cincinnus meristem produces one or two flowers (Figure 3G,I) [28]. The handedness of the cincinni varies among inflorescences. Usually it is sinistrose (left-handed), with the secondary bract and flower forming to the left in a polar view (Figure 3G). Occasionally (frequency < 1%), it can be dextrorse (right-handed), with the secondary bract and flower forming to the right.

The *Canna* flower is trimerous and consists of three sepals (S) in the outermost whorl followed concentrically by a whorl comprising three petals (P), two whorls comprising three androecial members (stamens and staminodes) each, and a trilocular ovary. The flower is predominantly asymmetric due to the differential distribution of abortion and petaloidy among members of the androecium. In the outer androecial whorl, only the lateral staminode (So1) fully develops into a mature organ; the abaxial member (So2) always aborts while the adaxial member (So3) can vary in its developmental trajectory (Figure 3H) [5]. In the inner androecial whorl, the adaxial androecium member (FS) develops a fertile anther (A) and a petaloid appendage (PA; Figure 3C,F) and is itself asymmetric in form. The lateral two androecium members develop into an inner petaloid staminode (Si) and a labellum (L; Figure 3H), the latter of which is also slightly asymmetric. A three locular ovary develops at the center of the flower. The style is laminar with asymmetric placement of the stigmatic surface, further contributing to overall floral asymmetry (Figure 3B,E).

### 3.2. Morphology of the Partial Florescence and Flower Structure in Aberrant Collections

**Figure 4:**
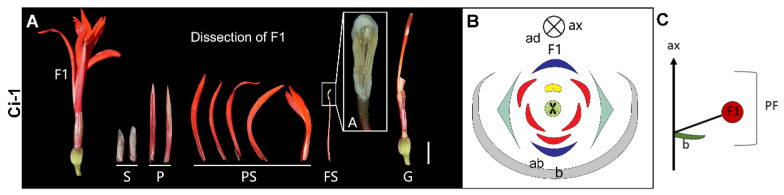
Single flower. (**A**) Photograph and (**B**) diagram of the partial inflorescence of Ci-1. (**C**) Longitudinal scheme of the lateral single flower. Bar = 1 cm.

**Figure 5:**
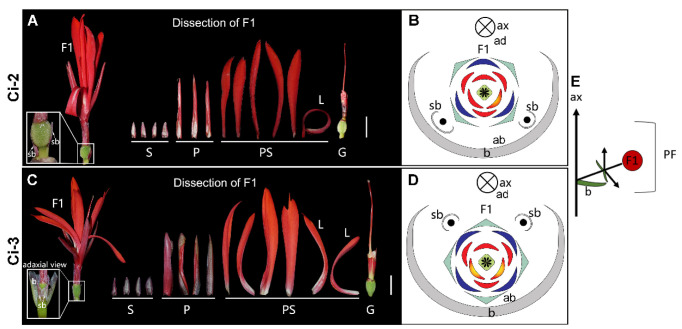
1-flowered cyme. (**A**) Photograph and (**B**) diagram of the partial inflorescence of Ci-2. (**C**) Photograph and (**D**) diagram of the partial inflorescence in Ci-3. (**E**) Longitudinal scheme of the 1-flowered cyme. Bars = 1 cm.

**Figure 6:**
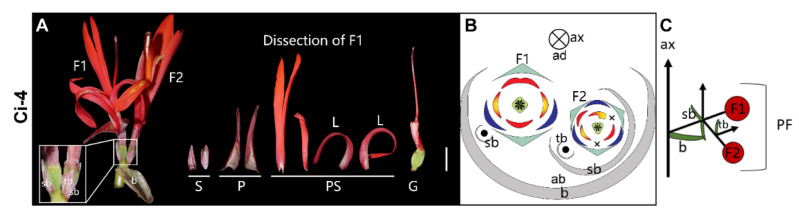
2-flowered cyme. (**A**) Photograph and (**B**) diagram of the partial inflorescence of Ci-4. (**C**) Longitudinal scheme of the 2-flowered cyme. Bar = 1 cm.

**Figure 7:**
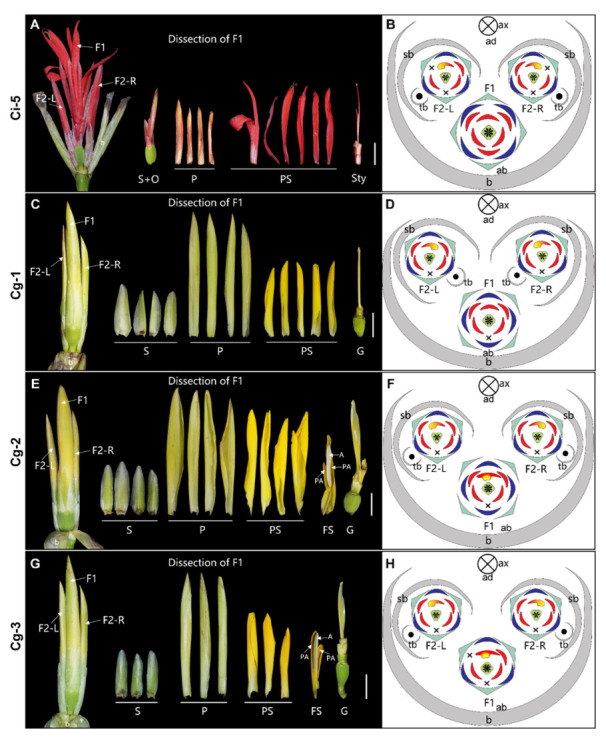

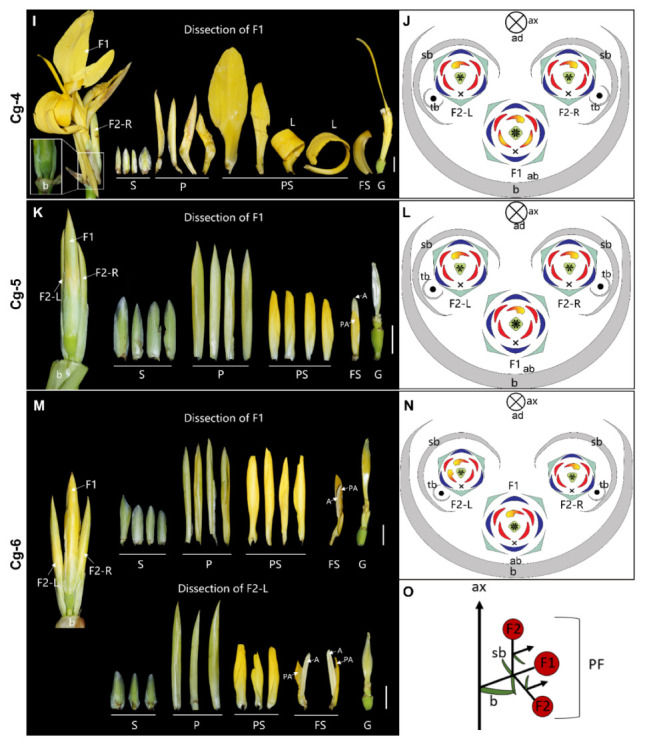
3-flowered cyme. (**A**) Photograph and (**B**) diagram of the partial inflorescence of Ci-5. (**C**) Photograph and (**D**) diagram of the partial inflorescence of Cg-1. (**E**) Photograph and (**F**) diagram of the partial inflorescence of Cg-2. (**G**) Photograph and (**H**) diagram of the partial inflorescence of Cg-3. (**I**) Photograph and (**J**) diagram of the partial inflorescence of Cg-4. (**K**) Photograph and (**L**) diagram of the partial inflorescence of Cg-5. (**M**) Photograph and (**N**) diagram of the partial inflorescence of Cg-6. (**O**) Longitudinal scheme of the 3-flowered cyme. In unopened flowers, the characteristics defining the labellum have not developed, thus the labellum’s position is not indicated in corresponding figures (**C**–**H**,**K**–**N**). F2-L, the left secondary flower; F2-R, the right secondary flower. Bars = 1 cm.

**Figure 8:**
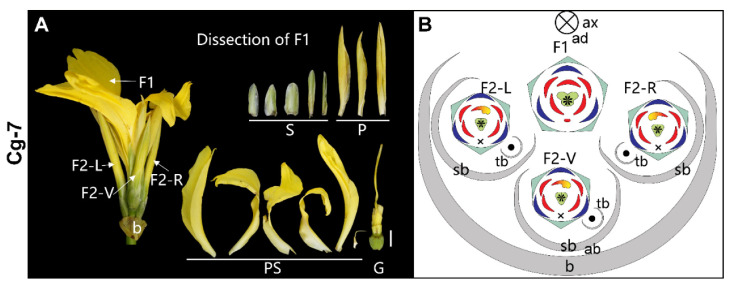
4-flowered cyme. (**A**) Photograph and (**B**) diagram of the partial inflorescence of Cg-7. F2-V, the ventral secondary flower. Bar = 1 cm.

**Figure 9:**
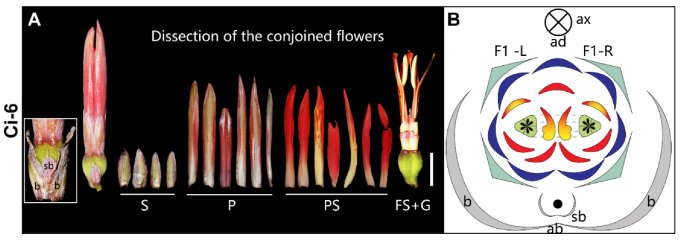
2-flowered thyrse. (**A**) Photograph and (**B**) diagram of the partial inflorescence of Ci-6. Dashed line in (**B**) indicates the ovaries are fused. Bar = 1 cm.

**Figure 10:**
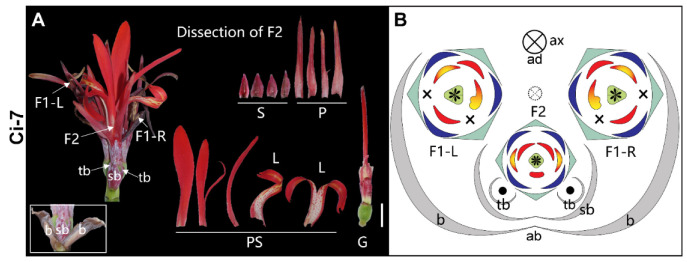
3-flowered thyrse. (**A**) Photograph and (**B**) diagram of the partial inflorescence of Ci-7. Dashed circle with the × in (**B**) indicates the early aborted raceme axis of the truncated thyrse. Bar = 1 cm.

**Figure 11:**
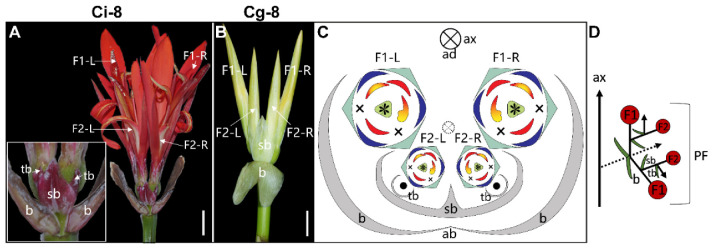
4-flowered thyrse. The partial inflorescence photographs of (**A**) *C. indica* collection Ci-8, and (**B**) *C. glauca* collection Cg-8. (**C**) The partial inflorescence diagram for both Ci-8 and Cg-8. (**D**) Longitudinal scheme of the 4-flowered thyrse. Dashed circle with a × in (**C**) and the dotted arrow in (**D**) indicate the early aborted raceme axis of the truncated thyrse. Bars = 1 cm.

The partial florescence structures among the aberrant collections are different from a typical *Canna* cincinnus and can be grouped into three main forms: **1. Single flower**—the simplest state of a partial inflorescence which results from a reduction in the typical cincinnus (Figure 4); **2. Cyme**—one flower formed at the first order, followed by two or three flowers to form a dichasium or a trichasium, which reverts back to monochasial at higher orders (Figure 5, Figure 6, Figure 7 and Figure 8); **3. Thyrse**—paired flowers subtended by conjoined primary bracts, likely representing a short raceme at the first order with each flower forming the primary flower of a cincinnus. The thyrse produces two, three, or four mature flowers in total depending on the length of the cincinni (Figure 9, Figure 10 and Figure 11). The floral morphology of each collection (Ci-1-8, Cg-1-8; Table 1) is described according to this classification of partial inflorescence structures.

**Single flower** (Figure 4)—In our only collection of a single flower (Ci-1), no secondary bract was detected (Figure 4A,C). The flower is zygomorphic, with two sepals, two petals, three fully developed outer petaloid staminodes, two inner petaloid staminodes, a dorsal fertile stamen with two thecae and a filamentous filament, and two carpels (Figure 4A,B). The style is only slightly laminar.

**1-flowered cyme** (Figure 5)—This partial inflorescence has dichasial branching in the first order, with only the primary flower fully developed (Figure 5E). Two secondary bracts (sb) are located on the lateral sides of the primary flower and can be either more abaxial (Ci-2) or more adaxial (Ci-3) in placement. In Ci-2, the primary flower has four sepals and three petals (4+3) instead of the normal 3 + 3 condition (Figure 5A,B); whereas, in the flower of Ci-3, the sepal and petal numbers are all increased to four (4+4; Figure 5C,D). The flowers in both collections are zygomorphic and lack a fertile (anther-bearing) stamen, with six petaloid staminodes dominating the floral display. A four-locular ovary develops at the center of the flower, and the style is filamentous instead of laminar (Figure 5A–D).

**2-flowered cyme** (Figure 6)—The 2-flowered cyme starts off as dichasial, with two secondary bracts (sb) produced laterally on both sides of the primary flower (Figure 6C). In the collection (Ci-4), the primary flower (F1) and secondary flower on the right side (F2) develop into mature flowers, while the left-side secondary flower aborts (Figure 6C). In the secondary order branch, the inflorescence becomes monochasial, possessing only a single tertiary bract (tb) that appears on the left abaxial side of the secondary flower (Figure 6A–C). The primary flower is zygomorphic with two sepals, two petals, and one whorl of four petaloid staminodes. Both lateral staminodes are curved and spotted like labella. The adaxial staminode is full developed to a bifid structure. The ovary has four locules, and the style is filamentous rather than laminar. The secondary flower in Ci-4 is an asymmetric flower with normal (dextrorse) morphology.

**3-flowered cyme** (Figure 7)—The primary flower (F1) and the two secondary flowers (F2) in the first order dichasium are fully developed, resulting in three mature flowers forming the partial inflorescence (Figure 7O). A single tertiary bract (tb) is associated with each secondary flower, indicating that the cyme becomes monochasial after one round of dichasial branching. Among all the 3-flowered cymes observed, the primary flower (F1) at the center of the partial inflorescence is variable in floral organ number, stamen fertility, and floral symmetry (Figure 7). With the exception of Cg-3, the number of sepals and petals is increased to four. The carpel number of each collection is four. The primary flower is zygomorphic in Ci-5 and Cg-1, with the dorsal stamen fully petaloid (Figure 7A–D). In Cg-2 and Cg-3, the dorsal fertile stamen has petaloid appendages on both sides of the anther, a unique feature that can be regarded as a transition stage which makes the primary flower not fully zygomorphic (Figure 7E–H). In Cg-4, Cg-5, and Cg-6, the dorsal stamen is a half-fertile stamen with an appendage and the flower is asymmetric as in normal flowers (Figure 7I–N). The style is filamentous in Ci-5, Cg-1, and Cg-4 (Figure 7A,C,I) but retains the typical laminar form in Cg-2, Cg-3, Cg-5, and Cg-6 (Figure 7E,G,K,M).

The two lateral secondary flowers in 3-flowered cyme have the same phenotype as normal *Canna* flowers: they are asymmetric and mirror images of one another (Figure 7B,D,F,H,J,L). In the case of Cg-6, the inner left petaloid staminode in the left secondary flower (F2-L) transforms to a fertile stamen that appears identical to the typical fertile stamen form (Figure 7M,N).

**4-flowered cyme** (Figure 8)—In accession Cg-7, three secondary flowers (F2-L, F2-R, and F2-V) are produced on the left, right, and abaxial side of the primary flower to form a 4-flowered cyme (Figure 8A). Each of the three secondary flowers has a tertiary bract (tb), indicating that they form a monochasium, although the third order flowers are all aborted (Figure 8B). The primary flower (F1) is zygomorphic with the presence of five sepals, three petals, and six petaloid staminodes among which the outer abaxial one is quite small. The ovary has four locules; the two adaxial locules are larger than the two abaxial locules making the ovary zygomorphic. The style is symmetrical and filamentous. The three secondary flowers of this cyme have the normal (wild type) structure (Figure 8A,B).

**2-flowered thyrse** (Figure 9)—In Ci-6, a pair of conjoined flowers are subtended by two fused primary bracts (Figure 9A). This conjoined structure is supposed to be derived from a truncated thyrse with two opposite cincinni, each comprising a single mature flower (Figure 9B). The conjoined flowers share four sepals and seven petals and the two adaxial petals are fused. There are seven petaloid staminodes, two of which are labella. Two fertile stamens are located adjacent to the midline of the floral complex as delineated by the axis of the short raceme (Figure 9B) with their corresponding anthers (each with a single theca) located on the abaxial side of the floral pair and the petaloid appendages on the adaxial side (relative to the main florescence axis). Two trilocular ovaries are fused, but the two styles remain separated and are laminar as appear in normal *Canna* flowers (Figure 9A,B). 

**3-flowered thyrse** (Figure 10)—In Ci-7, a pair of primary flowers (F1-L and F1-R) form, subtended by two fused primary bracts (Figure 10A). They are asymmetric and mirror images of one another. The fertile stamens of the floral pair are located adjacent to the midline of the floral complex (Figure 10B). Each primary flower has a corresponding secondary bract formed on the abaxial side and fused to one another (Figure 10B). The only secondary flower (F2) is subtended by two fused secondary bracts and has two tertiary bracts forming on its sides. The secondary flower is zygomorphic, with four sepals, four petals, and five petaloid staminodes including two lateral labella. The ovary has four locules and the style is symmetric and filamentous (Figure 10A,B). 

**4-flowered thyrse** (Figure 11)—In Ci-8 and Cg-8, we observe two opposite cincinni subtended by the two primary bracts leading to a total of four mature flowers; two primary and two secondary (Figure 11A,B). The two primary flowers are affiliated with a single secondary flower each, and each secondary flower is subtended by a secondary bract (Figure 11D). The two secondary bracts are fused in both Ci-8 and Cg-8, and two tertiary bracts are found on the abaxial sides of the two secondary flowers. All the flowers are asymmetric with only a half fertile stamen plus appendage as in normal *Canna* flowers. The fertile stamen within each flower is located adjacent to the midline (Figure 11C).

### 3.3. The Form of the Fertile Stamen Is Correlated with the Placement of Higher Order Bracts

With the exception of the 2-flowered and 4-flowered thyrses which bear double the number of normal flowers, the flowers born on the aberrant florescence axes described above all have zygomorphic flowers resulting from a variation in merosity (number of mature floral organs; Figure S2) and symmetry of typically asymmetric floral organs. 

The number of fertile stamen or the degree of stamen petaloidy appears to be a main factor impacting the symmetry of a *Canna* flower. Among all the normal and aberrant *Canna* flowers we observed, the number of thecae produced per flower varies from 0 (no fertile stamen) to 1 (a ½ fertile stamen) or 2 (a symmetrical fertile stamen). The fertile stamen, if present, is part of the inner androecial whorl on the dorsal side of the flower. In the case of “paired flowers” (Figure 7B, Figure 8B and Figure 9C), “dorsal” is relative to the midline of the partial florescence. The ability of the inner dorsal stamen to be fertile (two thecae), half fertile/half petaloid (one theca) or fully petaloid (no theca) is unique to Cannaceae and Marantaceae and plays an important role in controlling floral asymmetry in both families.

**Figure 12:**
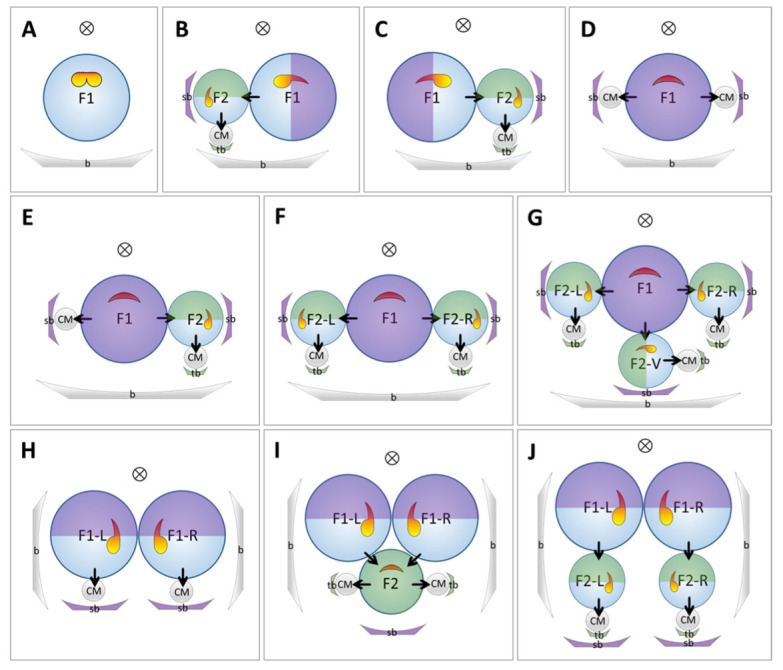
The correlation between the adaxial stamen form and the next order inflorescence branches. (**A**) Single flower (Ci-1); (**B**) Sinistrose cincinnus (normal type); (**C**) Dextrorse cincinnus; (**D**) 1-flowered cyme (Ci-2 and Ci-3); (**E**) 2-flowered cyme (Ci-4); (**F**) 3-flowered cyme (Ci-5); (**G**) 4-flowered cyme (Cg-7); (**H**) 2-flowered thyrse (Ci-6); (**I**) 3-flowered thyrse (Ci-7); (**J**) 4-flowered thyrse (Ci-8 and Cg-8). Small arrows within a partial inflorescence indicate the developing sequence of the flowers. The half in the primary flower distal to the secondary bract is shaded with purple, and the half in the secondary flower distal to the tertiary bract is shaded with green.

By characterizing the position of the laminar, petaloid outgrowth of the fertile stamen in each observed collection (Figure 12), we found a general rule for stamen petaloidy with respect to overall floral position within the inflorescence. In all but one case, the fertile stamen is orientated with respect to the next higher order bract, and the petaloid appendage is always located on the half of the dorsal stamen that is distal to the next higher order bract. For the primary flower (F1), the next order bract determining stamen orientation is the secondary bract (sb) (see Figure 12; purple). For the secondary flower (F2), the next order bract determining stamen orientation is the tertiary bract (tb) (see Figure 12; green). If there is no higher order bract, as in the single flower, the stamen is fully fertile and the flower is zygomorphic (Figure 12A). If there is a single higher order bract (i.e., in a monochasium), then the half of the dorsal stamen distal to the higher order bract is petaloid, whereas the other half proximal to the bract develops into the fertile anther (cf. orientation of ½ fertile stamen in Figure 12B,C). If there are two higher order bracts on both left and right sides of the flower, the stamen will be fully petaloid and no thecae will form. This rule is consistent in the primary flower of 1-flowered cymes (Figure 12D), in 2-flowered cymes (Figure 12E) and in some cases of 3-flowered cymes (Figure 12F) as well as in the secondary flower of 3-flowered “flower pair/double cyme” (Figure 12I). The stamen is also fully petaloid if there are three higher order bracts. In this case, both lateral sides of the flower are occupied by higher order bracts forming a dichasium as shown in the primary flower of a 4-flowered cyme (Figure 12G).

### 3.4. A Proposed Model to Explain the Correlation of in Florescence Architecture and Floral Symmetry

Floral symmetry is an emergent property, observable throughout the development of floral organs and ultimately responsible for generating a flower’s mature form. Although most *Canna* flowers are asymmetric due to the differential formation of organs in the androecial and gynoecial whorls, the occurrence of aberrant flowers provides insights into the various developmental processes and constraints that may be involved in generating overall patterns of floral symmetry. Observations of naturally occurring aberrant flowers in Cannaceae has enabled us to hypothesize certain rules of development that may more broadly apply to the formation of floral symmetry and, perhaps more importantly, floral asymmetry.

**Figure 13:**
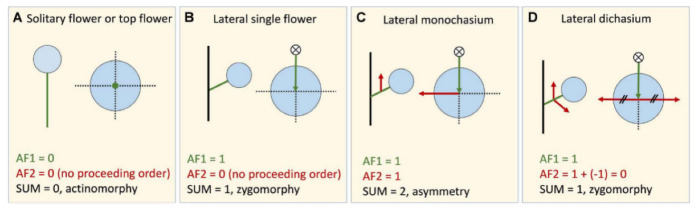
Explanatory model for the floral symmetry influenced by the inflorescence structure. (**A**) Solitary flower or top flower, AF1 = 0, no preceding order or AF2 = 0, the sum of two asymmetry factors is 0, usually the floral symmetry is actinomorphy. (**B**) Lateral single flower, AF1 is 1, and no proceeding order/flower, AF2=0, the sum of two asymmetry factors is 1, the flower tends to be zygomorphic. (**C**) Lateral monochasium, AF1 is 1, AF2 is 1, the flower has two asymmetry factors with different directions. SUM_AF_ is 2. This explains the floral asymmetry in Cannaceae. (**D**) Lateral dichasium, AF1 is 1, as it produced two branches/flowers in opposite directions, the asymmetry factors of proceeding order offset each other, AF2 is 0. The flower has SUM_AF_ = 1 along dorsal-ventral direction and theoretically is dorsal-ventrally zygomorphic. AF1, asymmetry factor produced by the preceding order, indicated by green arrow; AF2, asymmetry factor produced by the proceeding (next) order, indicated by red arrow.

The final symmetry of a flower can be predicted by how the flower is situated in the inflorescence. Floral symmetry is impacted by the position of the flower in the inflorescence when the floral primordium is generated (i.e., influenced by its position relative to the current order) and as it develops (i.e., influenced by its position relative to the next order). Although the genetic or molecular mechanism of this influence on the floral meristem remains unknown, here we generally call it “asymmetry factor” or AF. The potential rule can be simplified as a vector additive operation of the asymmetry factors produced by the preceding order (AF1) and proceeding order (AF2). Because the symmetry of a flower is generally defined from an “en face” view at anthesis [14], only transverse developing direction will contribute to the asymmetry factor to the flower, while vertical developing direction will not influence the floral symmetry. For example, if the current order is a solitary flower or top flower, the floral primordium is produced from a vertical direction; this does not contribute to the asymmetry factor influencing the floral form (Figure 13A) and we define AF1 = 0. For all lateral flowers, AF1 = 1 (Figure 13B–D). AF2 is the asymmetry factor produced by the proceeding order of the flower and is determined by the presence of net direction exerted by the lateral branches. If the proceeding order is one flower, monochasium, or raceme (i.e., there is one transverse net direction; Figure 13C), then AF2 = 1; if the proceeding order is no flower, dichasium or other branching forms that can offset each other on the transverse direction resulting in net direction (Figure 13B,D), AF2 = 0. The overall symmetry of a floral can be predicted by calculating the vector sum of these two asymmetry factors: SUM_AF_ = |AF1 + AF2| (notice that the directions of AF1 and AF2 should be taken into consideration).

In general, if SUM_AF_ = 0 the flower tends to be actinomorphic (Figure 13A); if SUM_AF_ = 1, the predicted floral symmetry is zygomorphy (Figure 13B,D); and if SUM_AF_ = 2, the flower is likely to be asymmetric (Figure 13C). This proposed model explains why the solitary flower is usually actinomorphic while zygomorphic flowers are typically found in a lateral position (Figure 13A,B). Furthermore, it also provides insights into the physical mechanisms underlying the development of the asymmetric flower. The asymmetric flower is correlated with the asymmetric surroundings in which it developed, as seen with the lateral cincinnus of *Canna*. A similar phenomenon was observed in *Tradescantia* (Commelinaceae), the flower of which is arranged in a monochasial partial florescence and has a transient slight asymmetry in its early developmental stages [3].

All flowers observed in this study, including normal and aberrant *Canna* flowers, follow this proposed model (see Figure S3 and Table S1). Whether the model can be universally applied to understand floral symmetry and asymmetry across Angiosperms remains to be tested.

## 4. Discussion

### 4.1. A Possible Cause of Floral Symmetry Variation in the Primary Flowers of 3-Flowered Cymes

The floral symmetry varies among the primary flowers in 3-flowered cymes. In some cases (Figure 7A–D), primary flowers are zygomorphic and the dorsal stamen is fully petaloid. Some flowers, however, display an intermediate condition in which the dorsal stamen comprises one theca and two petaloid appendages (Figure 7E–H). A similar morphology with stamens that are both fertile and petaloid can be found in Costaceae [e.g., *Costus spicatus* (Jacq.) Sw.] [29] and some species of Zingiberaceae [e.g., *Alpinia oxyphylla* Miq., *Kaempferia rotunda* L. and *Zingiber zerumbet* (L.) Sm]—closely related families to the Cannaceae. In other cases of aberrant *Canna* flowers (Figure 7I–N), the dorsal stamens of primary flowers remain half fertile and half petaloid, and the flowers are asymmetric as in normal *Canna* flowers. We propose that the timing of floral organ development with respect to the flower’s position in the inflorescence accounts for this variation. When a second-order branch begins to emerge, the floral organs of the primary flower have not yet differentiated (with the exception of the first sepal; as shown in Figure 3G). This indicates that during the development of normal *Canna* flowers, timing is such that development of the second-order inflorescence unit overlaps significantly with the organ-forming stages of flowers in the previous order. This may lead to physical constraints or molecular interactions (e.g., gene expression or protein interactions) between the primary flower primordium and the cincinnus meristem (and between the primary flower and the secondary inflorescence unit), leading to the asymmetrical development of the flower. If the proceeding order branch is delayed in growth, it may have less impact on the development of the primary flower. For example, the dichasium in 3-flowered cymes are likely to be alternate rather than opposite such that the two secondary branches do not emerge simultaneously. As a result of this timing effect, the degree of the stamen petaloidy varies in the primary flowers of 3-flowered cymes.

### 4.2. Comparison of Paired Flowers between Marantaceae and Aberrant Collections of Cannaceae

Cannaceae and Marantaceae present a uniquely derived floral structure within the Zingiberales. Species in both families produce asymmetric flowers with the asymmetry manifested by the presence of a half-fertile stamen (Figure S4A). The partial florescence in Marantaceae comprises paired flowers that are mirror images of one another. Comparative morphological studies show that the “flower pair” is actually a reduced thyrse, and each flower represents a reduced cincinnus [30,31]. Some *Canna* aberrant samples (Figure 9, Figure 10 and Figure 11) also bear paired flowers that are mirror images that are—to some extent—similar to the flower pairs found in Marantaceae. The structures of flower pairs in *Canna* aberrant cases and in typical Marantaceae are compared in Figure S4B. In Cannaceae, both primary bracts and secondary bracts are present, whereas in Marantaceae, the primary bracts that subtend the single-flowered cincinnus are usually missing and the secondary bracts are only occasionally present (e.g., in some species of *Calathea* G.Mey.). The similarity between Cannaceae and Marantaceae paired flowers suggests that the petaloid and asymmetric stamen in these two families is homologous and derived from the shared common ancestor.

### 4.3. Studying Aberrant Flowers as an Approach to Understanding Normal Floral Morphology

The development of flowers is a precisely regulated process. Many factors, including gene expression, hormone production, physical stress, and environmental variation work together and interact with each other to form a regulatory network. Alterations to the floral primordium may induce change in the entire developmental sequence and produce an aberrant flower pattern, providing insights into both evolutionary and developmental processes [32,33,34]. The emergence of aberrant flowers can be used for evolutionary interpretations of developmental patterning as well as determining the role of specific gene function through genetic studies [35]. In 1744, Linnaeus described a mutant *Linaria vulgaris* Mill. with radially symmetric flowers which he termed a ‘peloria’ [36]. Characterized as naturally occurring mutants by Darwin, the peloric flowers in *Antirrhinum* were later used to decode the genetic basis underlying floral symmetry development [37], demonstrating the importance of naturally occurring terata in exploring the patterns and processes that underly floral development and drive the evolution of floral diversity. 

In Zingiberales, most records of aberrant flowers focus on species in the Zingiberaceae and include attempts to reveal the morphological nature and/or homologies of particular floral organs. Rao [38] reported aberrant flowers in six species of *Alpinia* Roxb., *Hedychium* J.Koenig, *Kaempferia* L. and *Curcuma* L. with aberrant phenotypes in merosity and/or the position of the fertile stamen, the ovary locules, and the nectar glands. Maas [39] found an aberrant flower of *Renealmia goyazensis* K.Schum. & Gagnep., in which two fully developed anthers replaced the lateral lobes of the labellum. A similar flower was described for a population of *Z. zerumbet* growing on the north shore of Kauai (Baker R. & Bartlett M., personal communications). Chen and Wu [40] found an aberrant flower with two fertile stamens and one subulate appendage in *A. guangdongensis* S. J. Chen et Z. Y. Chen, and they regarded it as an atavistic mutation with one of the subulate appendages replaced by a fertile stamen. Song et al. [41] described six types of abnormal flowers in *Alpinia*; these abnormalities include flowers with 2 fertile stamens, 1.5 stamens, 1 stamen, single stamen with only one theca (½ stamen), and fertile stamen completely absent as well as the occurrence of twin flowers forming mirror images. Li et al. [42] recently reported two-staminate aberrant flowers in a cultivar *A. intermedia* ‘shengzhen’. These aberrant flowers share with one another alterations in the form of the androecial members that indicate common deviations from a canalized developmental pathway. Given the apparent lack of heritability of these aberrant flowers (including those in this study), these variations in phenotype are not likely due to genetic mutations but rather arise as a result of environmental influences during development. The Zingiberales families have undergone an evolutionary reduction in the fertile stamen number from an ancestral condition of (6)5 (Musaceae) to 1 (Costaceae, Zingiberaceae) and 1/2 (Cannaceae, Marantaceae) [43,44,45,46]; however, the developmental mechanism of this transition remains unknown. Studying aberrant flowers among the Zingiberales is an approach that helps to characterize developmental shifts leading to differential expression of petaloidy and fertility among androecial members and thus can provide insights into the evolution of floral organs across the order.

## 5. Conclusions

By dissecting and documenting the merosity, organ position, symmetry, and position in the inflorescence of specimens with aberrant flowers, we conclude that the formation of the asymmetric flower of *Canna* is strongly correlated with its position within the inflorescence. In normal *Canna* inflorescences, the flower is a terminal unit within a thyrse. Transition of the partial florescence from the normal cyme results in alterations in merosity, floral organ identity, and overall floral symmetry in the flowers born by that partial florescence. Within *Canna*, the rule that the petaloid half of the fertile stamen is consistently elaborated distally to the higher order bract may provide insights into the structural and developmental rules governing the origin and elaboration of asymmetry in flowers.

## Figures and Tables

**Table 1:** List of aberrant flower collections investigated.

Collection ID.	Species	Partial Inflorescence	Figure
Ci-1	*Canna indica*	Single flower	4, 12A
Ci-2	*Canna indica*	1-flowered cyme	5A,B,E, 12D
Ci-3	*Canna indica*	1-flowered cyme	5C–E, 12D
Ci-4	*Canna indica*	2-flowered cyme	6, 12E
Ci-5	*Canna indica*	3-flowered cyme	7A,B,O, 12F
Cg-1	*Canna glauca*	3-flowered cyme	7C,D,O
Cg-2	*Canna glauca*	3-flowered cyme	7E,F,O
Cg-3	*Canna glauca*	3-flowered cyme	7G,H,O
Cg-4	*Canna glauca*	3-flowered cyme	7I,J,O
Cg-5	*Canna glauca*	3-flowered cyme	7K,L,O
Cg-6	*Canna glauca*	3-flowered cyme	7M–O
Cg-7	*Canna glauca*	4-flowered cyme	8, 12G
Ci-6	*Canna indica*	2-flowered thyrse	9, 12H
Ci-7	*Canna indica*	3-flowered thyrse	10, 12I
Ci-8	*Canna indica*	4-flowered thyrse	11A,C,D, 12J
Cg-8	*Canna glauca*	4-flowered thyrse	11B–D, 12J

## Data Availability

Not applicable.

## Supplementary Materials

The following supporting information can be downloaded at: https://www.mdpi.com/article/10.3390/plants11192512/s1, Figure S1: Parsimony tree showing the phylogenetical placement of *Canna indica* and *C. glauca*; Figure S2: Merosity in aberrant flowers. Average number of (A) sepal, (B) petal, (C) stamen and (D) carpel of aberrant flowers in different partial florescence types. (E) Variety of carpel numbers in *Canna* wild type and aberrant flowers. Red lines indicate the organ numbers in wild type *Canna* flower. Bar = 1 cm; Figure S3: Diagram explanation for the symmetry of normal and aberrant *Canna* flowers based on the proposed model; Figure S4: Comparison of flower pair between Cannaceae and Marantaceae. (A) The phylogenetic tree of Zingiberales shows the close relationship between Cannaceae and Marantaceae. (B) The diagrams of flower pairs in Cannaceae and Marantaceae; Table S1: Floral symmetry speculated by the inflorescence structure based on the proposed model.
