# Supplementary material for: Correlation between Inflorescence Architecture and Floral Asymmetry—Evidence from Aberrant Flowers in Canna L. (Cannaceae)
Source: Plants (Basel). 2022 Sep 26;11(19):2512. doi: 10.3390/plants11192512 (PMC9571657; doi:10.3390/plants11192512)
Supplement: Supplementary file 1 [file plants-11-02512-s001.zip › Table S1.pdf]

Table S1. Floral symmetry speculated by the inflorescence structure based on the proposed model

Flower Studied	Current Order	AF1	Proceeding Order	AF2	SUM	Symmetry
Primary flower in Single flower	raceme	1	no flower	0	1	zygomorphy
Primary flower in normal cincinnus	raceme	1	monochasium	1	2	asymmetry
Primary flower in 1-flowered cyme	raceme	1	dichasium	0	1	zygomorphy
Primary flower in 2-flowered cyme	raceme	1	dichasium	0	1	zygomorphy
Primary flower in 3-flowered cyme	raceme	1	dichasium	0	1	zygomorphy
Primary flower in 4-flowered cyme	raceme	1	trichasium	1	1*	zygomorphy
Primary flower in 2-flowered thyrse	raceme	1	monochasium	1	2	asymmetry
Primary flower in 3-flowered thyrse	raceme	1	monochasium	1	2	asymmetry
Primary flower in 4-flowered thyrse	raceme	1	monochasium	1	2*	asymmetry
Secondary flower in normal cincinnus	monochasium	1	monochasium	1	2	asymmetry
Secondary flower in 2- flowered cyme	dichasium	1	monochasium	1	2	asymmetry
Secondary flower in 3- flowered cyme	dichasium	1	monochasium	1	2	asymmetry
Secondary flower in 4- flowered cyme	trichasium	1	monochasium	1	2	asymmetry
Secondary flower in 3-flowered thyrse	monochasium	1	dichasium	0	1*	zygomorphy
Secondary flower in 4-flowered thyrse	monochasium	1	monochasium	1	2	asymmetry

* Note: the net direction of AF1 and AF2 are both on the dorsal-ventral axis, so that SUM = 1.
