# Supplementary figures and images for: Correlation between Inflorescence Architecture and Floral Asymmetry—Evidence from Aberrant Flowers in Canna L. (Cannaceae)
Source: Plants (Basel). 2022 Sep 26;11(19):2512. doi: 10.3390/plants11192512 (PMC9571657; doi:10.3390/plants11192512)

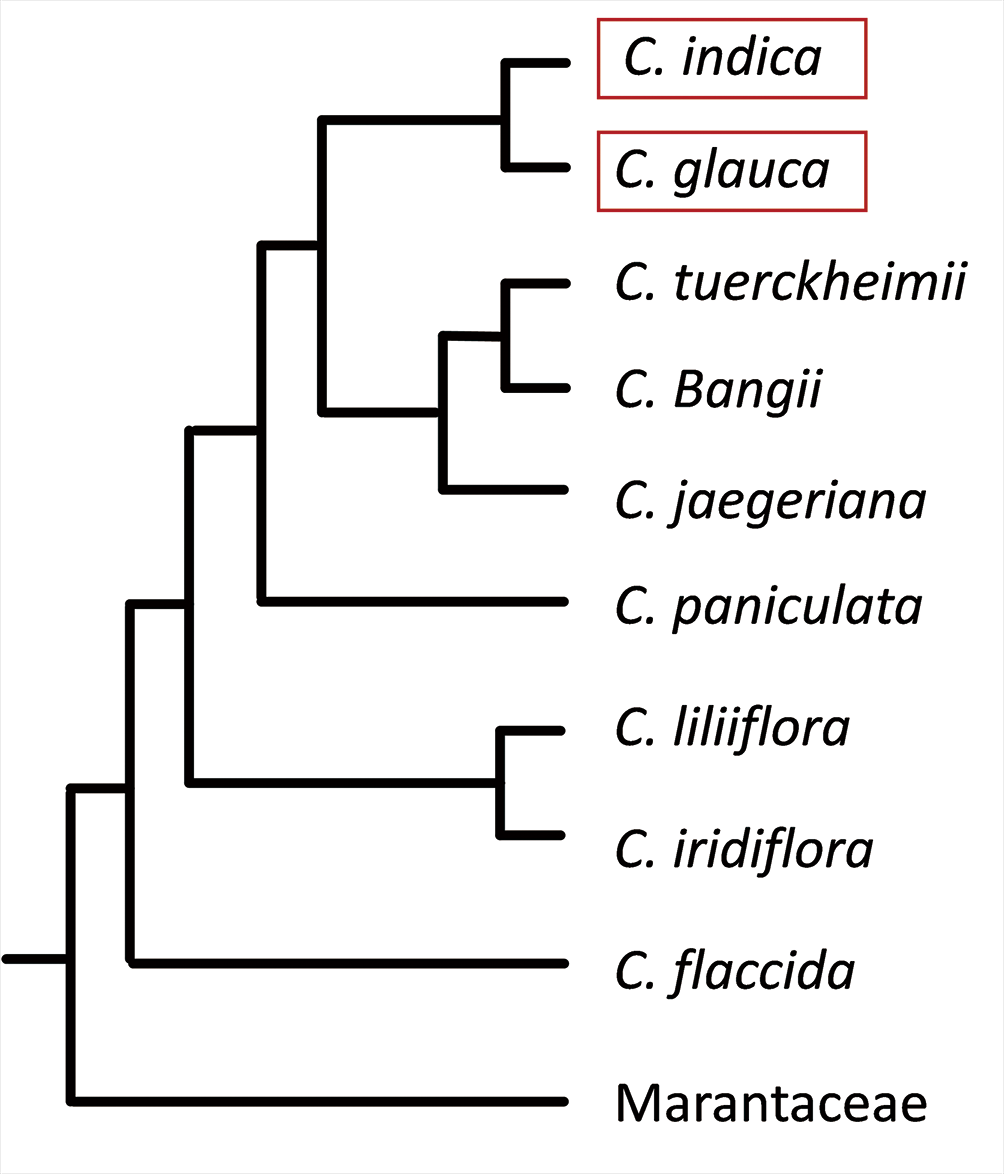

Supplement: Supplementary file 1 [file plants-11-02512-s001.zip › Figure S1.tif]

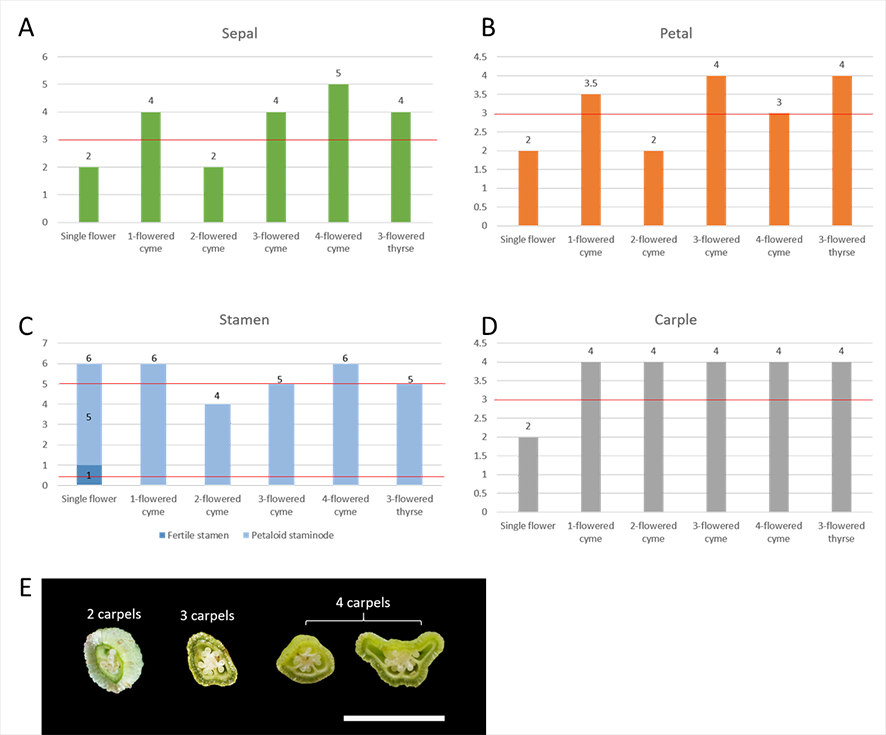

Supplement: Supplementary file 1 [file plants-11-02512-s001.zip › Figure S2.tif]

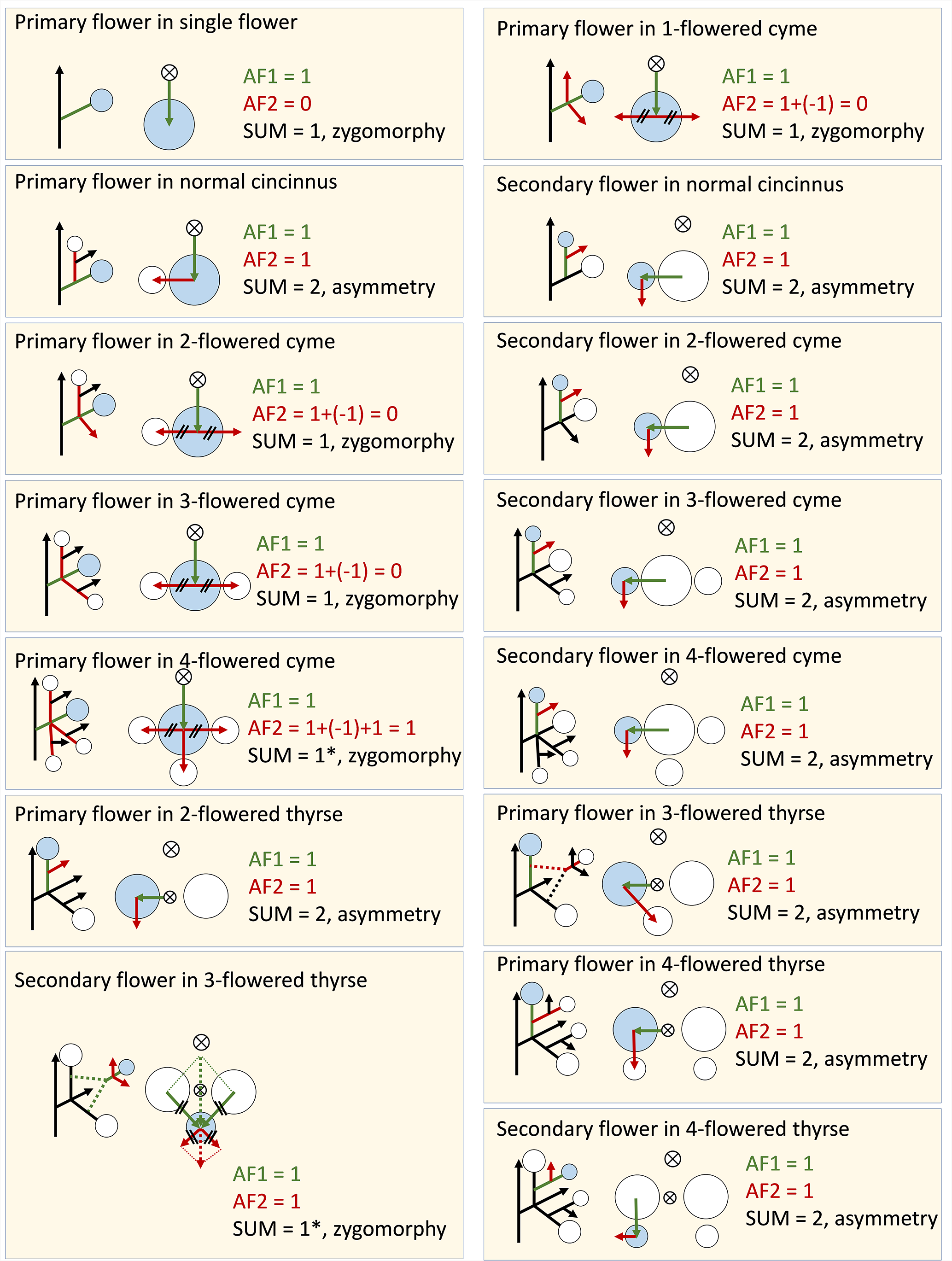

Supplement: Supplementary file 1 [file plants-11-02512-s001.zip › Figure S3.tif]

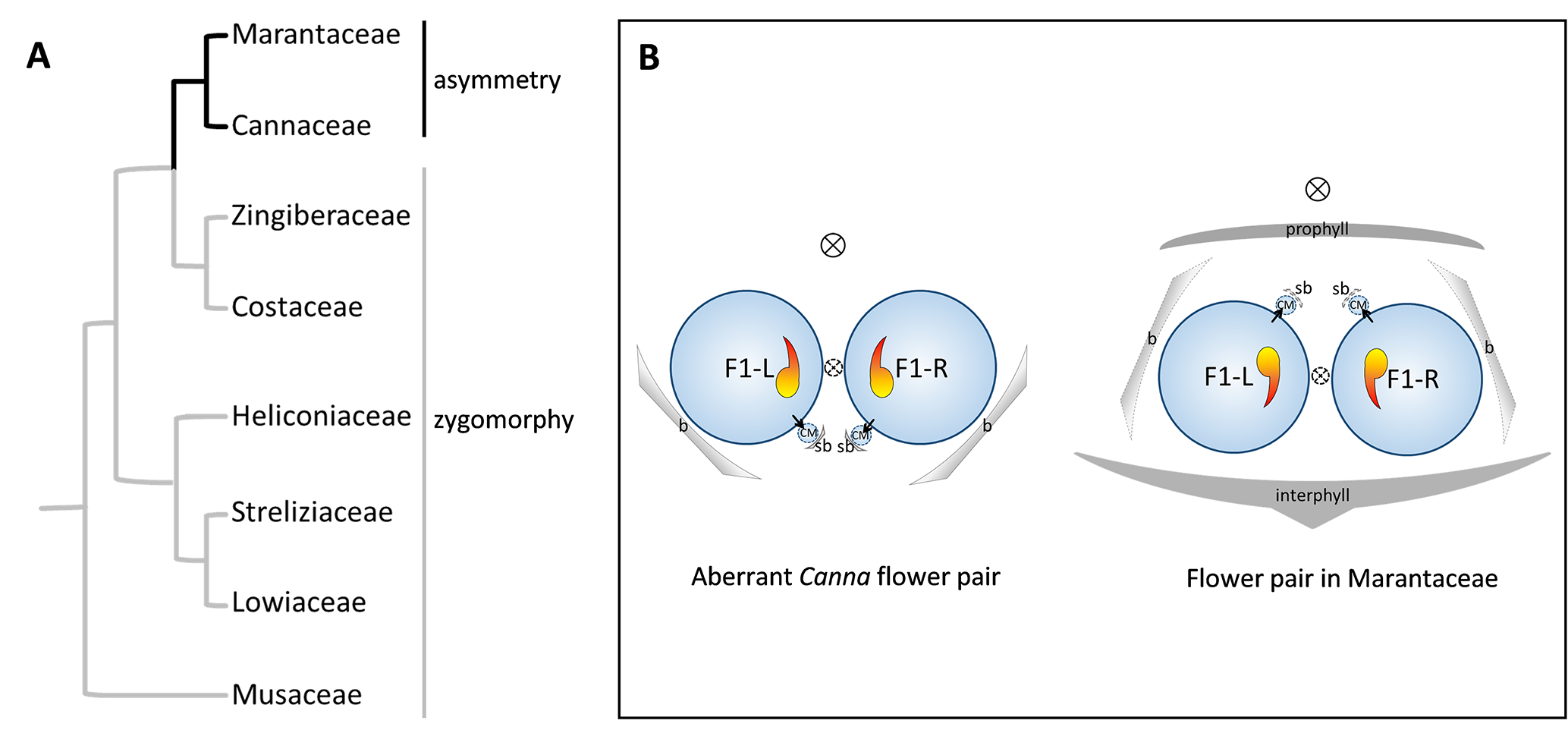

Supplement: Supplementary file 1 [file plants-11-02512-s001.zip › Figure S4.tif]
